# General Hospitals

**Published:** 1893-08-19

**Authors:** 


### The Institutional Workshop.

### HOSPITAL ADMINISTRATION.

### I.—GENERAL HOSPITALS.

Writing last year on this subject, we affirmed that those most interested in the administration of our voluntary hospitals have long desired to secure the adoption of some plan which should make it possible to compare results upon an identical basis. Years ago the difficulties in the way of making thorough comparisons between the relative success of various systems of administering medical institutions of the same size and importance were well-nigh insurmountable. Material was abundant, but each batch of facts had to be compiled and put together upon the plan favoured by the compiler. Hence it proved impossible to form a definite and convincing judgment of the reasons which caused one hospital to expend half as much again, or twice as much again per bed as another. Fortunately there was one factor at work which tended to build up the voluntary system. This was represented by a species of rivalry among the authorities as to which could prove itself most worthy of public support. Still, the best administered hospitals were at a disadvantage in the absence of reliable data, compiled upon an identical basis, by which the public would be prevented from forming a wrong judgment as to relative merits. In such circumstances the prize of liberal offerings sometimes fell, as it falls now, to the lot of institutions which are not among the best administered, although they may be the most successful in enlisting public sympathy by causing their work to be widely known.

Fortunately matters have improved in many directions during the last few years. The adoption of a uniform system of accounts, and its enforcement by the Committee of Distribution of the Hospital Sunday Fund, is tending to remedy the old views by making it more and more difficult for the relatively inefficient institutions to escape detection. The Council of the Metropolitan Sunday Fund has organised a system through its Committee of Distribution which enables any member of the public, by addressing a letter to the Lord Mayor at the Mansion House, to ascertain whether or no a particular hospital or medical charity is managed upon the most approved principles, and to what extent it worthily represents the highest form of administration at the time the inquiry is made. Hence there is no excuse for wealthy donors and others who wish to wisely give of their abundance for the alleviation of sickness amongst the poor. The enforcement of a regular system of audit by public accountants, in conjunction with the governors' auditor, is also tending to promote efficiency of the highest kind. In this connection we would earnestly impress upon all who speak upon hospitals to guard themselves from giving an impression, from any remark they may make, that the great hospitals, that is, hospitals which worthily represent that class of institution to which they belong, apart altogether from their size, are in any sense ill-managed at the present time. Speakers are too apt to generalise by declaring that there are good and bad hospitals. No doubt this is true, but only in the sense that the bad hospitals are relatively so few and insignificant in London to-day that they do but constitute the exception which proves the rule.

The last meeting of the Council of the Sunday Fund at the Mansion House proved that the Committee of Distribution are resolved to refuse grants to those hospitals which do not keep their books upon the uniform system, and employ public accountants as auditors. Those of our readers who bind up the numbers of The Hospital, will be able to estimate how important a declaration to this effect by Sir Sydney Waterlow in reality was, because they will see the attitude taken by the Council only a year ago, if they will refer to page 35 of our issue of August 27th, 1892. We may, perhaps, usefully insist that as the aim of The Hospital is to give, year by year, precise information and facts upon all points of interest in the administration of hospitals and kindred institutions, hospital governors, medical practitioners, and secretaries, will find it of the first importance to keep bound copies of each volume of The Hospital at hand for ready reference.

#### The Expenses of Management.

The Metropolitan Hospital Sunday Fund, as we stated last year, has for some years past prepared tables showing the number of beds occupied each year; the number of home visits from dispensaries; the number of cases treated, with approximate cost of treatment; the percentage of the cost of administration as compared with that of maintenance; the average amount of patients' payments; and the average gross annual income and expenditure during three years. This year the Council publishes in addition the percentage of the cost of administration to total expenditure. It is therefore possible to compare the working of each institution receiving a grant with every other institution on the list, and the results may be taken as evidence upon which thoughtful people may base the amount of money which they will give each year to various hospitals. In pursuance of our practice we now proceed to examine the percentage of the cost of administration to (*a*) that of maintenance, and (*b*) to total expenditure, as shown in these returns.

The table on the next page contains the names of all the general hospitals which have 400 beds and upwards, in addition to the London Homœopathic Hospital. A reference to page 365 of The Hospital for August 27th, 1892, will enable the reader to compare the progress made on the path of economy by each hospital included in the table. The reduction in the cost of management at King's College Hospital in 1892, as compared with 1891, amounts to upwards of 1½ per cent., a fact which should secure the immediate contribution by the public of the £50,000 for which an appeal was recently issued. Of the other hospitals, the London Hospital, Middlesex, St. Mary's, University College, Westminster, Charing Cross, Tottenham, West London, and the Homœopathic all show reduction in the cost of management, varying from 2¾ per cent. saved in the case of the last to under ½ per cent. On the other hand, Guy's, Seamen's, and the Royal Free show an increase varying from 1¼ per cent. in the case of Guy's to a small fraction at the last named hospital. St. George's occupies a place by itself, for, as we pointed out last year, we are of opinion that the Committee could with advantage spend a considerably larger sum than they do upon salaries, an item which, rightly understood, produces results, so far as efficiency in administration is concerned, in direct proportion to the percentage which it bears to a liberal and wise expenditure. We are glad to perceive that the Committee of St. George's have recently taken this advice to heart, and that the expenditure has risen from 4⅗ to a little over 5¼ per cent.

Many people have held that it is misleading to criticise the expenditure of the administration of a hospital on the basis of the cost of administration to that of maintenance. These critics urge that the only true basis is a percentage of the cost of administration to total expenditure. Being anxious to oblige all reasonable objectors we this year give a column showing the latter percentage of cost also. It will be seen, on comparing it with the percentage of cost to maintenance, that it makes a difference on an average of less than two per cent. in all cases, with the exception of University College, the Royal Free, and the Homœopathic Hospitals, where the difference is about one per cent. more. Apart from this consideration, no reasonable well-wisher of our hospitals can deny that the figures in the table are most encouraging to the public, and that they reflect the greatest credit upon the management all along the line. An inquirer in the *Standard* of the 10th inst. expressed a wish that steps might be initiated to secure increased economy in the charges of the various hospitals. We are enabled to present him and other critics with precise data worked out upon an identical basis, which proves that the Committees and other officers are fully alive to the importance of economy, and that they have succeeded in showing a reduction in these charges, although the year 1892 was, in many respects, probably the most trying which they have ever had to face.

We may add, for the information of those who have not made a study of hospital finance, that a careful investigatigation of the whole of the questions involved, based upon information which reveals the circumstances of all the great hospitals of the world, has brought us to the conclusion that the expenditure which it is essential that each Committee shall sanction, in order to secure the maximum of efficiency in the administration of a voluntary hospital, is represented by a maximum sum equal to 15 per cent. upon the total expenditure each year. This calculation relates to hospitals containing 100 beds and upwards. Judged by this standard, it will be seen that none of the general hospitals in London exceed this limit, and that the actual percentage of the cost of administration to the total expenditure is under 14 per cent. Bearing this in mind, and remembering the further fact that so many of the hospitals show a marked decrease in the percentage of cost during the year 1892, as compared with 1891, it is undoubtedly true that there are no public institutions in the world which approach the hospitals to which the charitable can give with the maximum of liberality, and an assured confidence that every penny of their contributions which it is possible to devote directly to the relief of the sick will be so devoted without reduction of any kind. Economy and efficiency are the watchwords of our voluntary hospitals, and that the principles which they embody are steadily enforced by the managers is proved to demonstration by the figures contained in the accompanying table.

Metropolitan General Hospitals.

| Name of Hospital. | Beds in Occupation. | Average Number Occupied. | Total In-patients Admitted. | Total Out-patients Admitted. | Percentage of cost of Administration to that of Maintenance. | Percentage of Administration to Total Expenditure. |
|---|---|---|---|---|---|---|
| London ... ... ... | 776 | 630 | 9,409 | 112,962 | 5·211 | 4·810 |
| Guy's ... ... ... ... | 600 | 433 | 6,136 | 59,846 | 8·924 | 8·192 |
| St. George's ... ... ... | 355 | 225 | 3,080 | 31,828 | 5·339 | 3·827 |
| Middlesex ... ... ... | 320 | 254 | 2,912 | 39,312 | 6·370 | 5·988 |
| St. Mary's ... ... ... | 281 | 246 | 3,729 | 30,975 | 6·513 | 6·113 |
| Seamen's ... ... ... | 253 | 219 | 2,690 | 13,745 | 12·182 | 10·797 |
| King's... ... ... ... | 220 | 184 | 2,212 | 24,265 | 10·929 | 9·852 |
| University ... ... ... | 207 | 183 | 2,799 | 36,999 | 14·139 | 12·387 |
| Westminster... ... ... | 205 | 186 | 2,989 | 26,202 | 6·498 | 6·090 |
| Charing Cross ... ... | 175 | 110 | 2,007 | 16,312 | 11·969 | 10·688 |
| Royal Free...... ... | 160 | 125 | 1,757 | 23,381 | 7·306 | 4·230 |
| German ... ... ... | 125 | 94 | 1,283 | 24,113 | 12·667 | 11·243 |
| Training (Tottenham) ... | 103 | 63 | 845 | 8,687 | 5·797 | 5·480 |
| West London ... ... | 101 | 92 | 1,620 | 26,089 | 11·441 | 10·267 |
| London Homœopathic ... | 94 | 53 | 761 | 10,245 | 16·057 | 13·831 |

